# Differentiation of Murine Bone Marrow-Derived Smooth Muscle Progenitor Cells Is Regulated by PDGF-BB and Collagen

**DOI:** 10.1371/journal.pone.0156935

**Published:** 2016-06-03

**Authors:** Clifford Lin, Yifan Yuan, David W. Courtman

**Affiliations:** 1 Ottawa Hospital Research Institute, Ottawa, Ontario, Canada; 2 Oregon Health and Science University, Portland, Oregon, United States of America; INSERM, FRANCE

## Abstract

Smooth muscle cells (SMCs) are key regulators of vascular disease and circulating smooth muscle progenitor cells may play important roles in vascular repair or remodelling. We developed enhanced protocols to derive smooth muscle progenitors from murine bone marrow and tested whether factors that are increased in atherosclerotic plaques, namely platelet-derived growth factor—BB (PDGF-BB) and monomeric collagen, can influence the smooth muscle specific differentiation, proliferation, and survival of mouse bone marrow-derived progenitor cells. During a 21 day period of culture, bone marrow cells underwent a marked increase in expression of the SMC markers α-SMA (1.93 ± 0.15 vs. 0.0008 ± 0.0003 (ng/ng GAPDH) at 0 d), SM22-α (1.50 ± 0.27 vs. 0.005 ± 0.001 (ng/ng GAPDH) at 0 d) and SM-MHC (0.017 ± 0.004 vs. 0.001 ± 0.001 (ng/ng GAPDH) at 0 d). Bromodeoxyuridine (BrdU) incorporation experiments showed that in early culture, the smooth muscle progenitor subpopulation could be identified by high proliferative rates prior to the expression of smooth muscle specific markers. Culture of fresh bone marrow or smooth muscle progenitor cells with PDGF-BB suppressed the expression of α-SMA and SM22-α, in a rapidly reversible manner requiring PDGF receptor kinase activity. Progenitors cultured on polymerized collagen gels demonstrated expression of SMC markers, rates of proliferation and apoptosis similar to that of cells on tissue culture plastic; in contrast, cells grown on monomeric collagen gels displayed lower SMC marker expression, lower growth rates (319 ± 36 vs. 635 ± 97 cells/mm^2^), and increased apoptosis (5.3 ± 1.6% vs. 1.0 ± 0.5% (Annexin 5 staining)). Our data shows that the differentiation and survival of smooth muscle progenitors are critically affected by PDGF-BB and as well as the substrate collagen structure.

## Introduction

It is becoming recognized that vascular smooth muscle progenitor cells (SMPCs) play important roles in vascular repair, remodelling and disease ([1, 2]). Some controversy remains over the relative contribution of SMPCs arising from the local vascular tissue versus those from the bone marrow [3–5], nonetheless, the application of bone marrow derived SMPCs has been shown to limit and stabilize atherosclerotic plaque development in ApoE-/- mice [2] and limit aneurysm progression in rats [6] and mice [7]. In addition, lesion progression in mice can be limited by systemic CXCL12 injections resulting in the recruitment of circulating SMPCs [8] and human studies demonstrate that circulating SMPC levels are reduced in acute coronary syndromes as compared to stable angina [2]. These studies suggest that bone marrow derived SMPCs may be important therapeutic targets that may be modified to favourably induce appropriate vascular remodelling.

SMCs exert complex effects in vascular disease: in normal arteries, SMCs are thought to be ‘silent’ and are harnessed during disease to remodel arteries in response to injury. With adaptive remodelling resulting in stabilized plaques by synthesizing a thick fibrous cap that protects against rupture. SMCs change from their physiological contractile phenotype to the pathophysiological synthetic phenotype and migrate into the intima where they advance disease progression by importing lipid, stimulating inflammation, producing extracellular matrix, and secreting matrix metalloproteinases [9–14]. The phenotypic alteration of SMCs is critically regulated by the matrix in atherosclerosis, with normal components of the healthy SMC matrix limiting modulation and atherosclerosis-associated degraded matrix proteins promoting phenotypic modulation [15]. Excessive collagen breakdown combined with inadequate synthesis weakens plaques thereby making them prone to rupture [16]. Monomeric collagen (non-fibrillar collagen) has been previously shown to reduce SMC contractility and stimulate inflammatory protein expression such as VCAM-1 and NF-kappaB [15, 17]. The monomeric collagen matrix also stimulates SMC proliferation in contrast to the reduction of cell growth on fibrillar collagen [17, 18]. These effects can be further enhanced when co-stimulated with platelet-derived growth factor (PDGF-BB) which possesses potent mitogenic and inflammatory effects and participates in ECM remodeling and has been shown to promote proliferation and differentiation in adjacent SMCs [19–21]

Establishing the origin of neointimal SMCs is central to understanding the pathogenesis of atherosclerosis however it currently remains elusive. It has been shown that a bone marrow cell population can readily differentiate into SMC-like cells depending on local environmental cues and proliferate and migrate to the neointimal SMC layer *in vivo* ([22], reviewed in [23, 24]). For example, transplanted bone marrow labelled with eGFP, lacZ, or by sex-mismatching has been shown to result in labelled recipient neointimal SMCs in the mechanical vascular injury, allograft vasculopathy and hyperlipidemia-induced models of atherosclerosis [3, 25–27].

In this study, we isolated murine bone marrow cells and differentiated them towards a smooth muscle lineage. We showed that the viability and capacity to maintain a differentiated phenotype of bone marrow derived SMPCs are strongly influenced by monomeric collagen and PDGF-BB, factors that are increased in atherosclerotic plaques. These results suggest that matrix remodelling and increased levels of PDGF-BB either in the plaque or remodelling vasculature microenvironment may limit SMCs involvement in healing atherosclerotic plaques *in vivo*.

## Materials and Methods

### Isolation of smooth muscle progenitor cells from mouse bone marrow

All animal procedures were carried out in accordance with the Canadian Council of Animal Care (CCAC) guidelines. the protocol (#573) was approved by the Animal Care Committee of St.Michael’s Hospital, Toronto. Female FVB/N mice were sacrificed at 6–10 weeks of age and bone marrow was collected from femorae and tibiae by aspiration. Harvested cells were dissociated and seeded into flasks at a density of 4 x 10^5^ cells/mm^2^ in α-MEM containing 10% FBS, 50 μM β-mercaptoethanol, 100 U/mL penicillin, and 100 μg/mL streptomycin (all from Invitrogen)(referred as 10% α-MEM throughout the manuscript). Cells were maintained in culture at 37°C in humidified 5% CO_2_.

As indicated, rat recombinant PDGF-BB (50 ng/mL, Sigma-Aldrich) was added directly to the culture media, alone or in combination with the receptor tyrosine kinase inhibitor AGL2043 (10 μM, Calbiochem) or a non-inhibitory control compound AG9 (10 μM, Calbiochem).

### Primary aortic smooth muscle cells

Primary aortic smooth muscle cells (SMCs) were isolated from an additional group of female FVB/N mice [28] as follows: a 2 cm length of aorta was resected from the mid-thoracic to the infrarenal region and placed in chilled DMEM containing 1% HEPES. Aortas were denuded of endothelium and adventitial layers by gentle scraping, chopped into 1 mm cubes, and incubated in DMEM containing 1% HEPES, 1% penicillin/streptomycin, collagenase type I (1.8 mg/mL), elastase type III (0.3 mg/mL), soybean trypsin-inhibitor type I (0.44 mg/mL), and BSA (2 mg/mL). After mechanical dispersion and incubation (1 h, 37°C), cell suspensions were diluted 25-fold in DMEM containing 10% FBS and antibiotics, centrifuged (652 *g*, 5 min), and resuspended and collected in the same media.

### RNA isolation and quantitative real-time PCR

RNA was extracted from mouse bone marrow and aortic tissue using RNeasy (Qiagen) and cDNA was synthesized (Omniscript RT, Qiagen). The expression of α-SMA, SM22-α, SM-MHC, and GAPDH mRNA were detected by qRT-PCR in triplicate assays using SYBR Green or TaqMan (Applied Biosystems) reactions. Details of the PCR and primer sequences are shown in Table 1. The pre-optimized SMMHC primer set (TaqMan, Mm00443013_m1) was purchased from Applied Biosystems).

**Table 1:** Primer sequences for SMC gene targets.

Gene	Forward Primer	Reverse Primer
α-SMA	5’-CGGCTTCGCTGGTGATGA-3’	5’-TCCCTCTCTTGCTCTGGGCTT-3’
SM-22-α	5’-CTCTAATGGCTTTGGGCAGTT-3’	5’-TGCAGTTGGCTGTCTGTGAAG-3’
GAPDH	5’-GCATGGCCTTCCGTGTTC-3’	5’-ATGTCATCATACTTGGCAGGCAGGTTTC-3’

### Immunolabelling and image analysis

Cells were fixed in 4% paraformaldehyde in PBS (w/v, pH 7.4) for 15 min, permeabilized in 0.1% Triton X-100 for 30 min, and blocked in 3% normal goat serum in PBS. Mouse anti-human smooth muscle actin IgG_2a_κ (1:200 in PBS/1% BSA, Clone 1A4, DAKO); rabbit anti-SM22 alpha antibody (1:1000 for both immunostaining and western blot, Abcam); rabbit anti-smooth muscle myosin heavy chain antibody (1:200, Abcam); mouse anti-vinculin (2 μg IgG_1_/mL, hVIN-1, Sigma); goat anti-mouse IgG-Alexa 488 (1:400, Molecular Probes) were used for immunostaining. F-actin was labelled with phalloidin-Alexa 546 (Molecular Probes), and nuclei were counterstained with TO-PRO-3 (2 μM, Molecular Probes).

Images were captured using a laser-scanning confocal fluorescence microscope (Bio-Rad Radiance) with a 40X objective (NA 1.3). To score expression of SMC markers, cells from each group were counted in 6 fields (minimum 500 cells in total). Cell morphology was quantified by tracing the outline of the cell as judged by cytoskeletal phalloidin staining and measuring the roundness (a value computed by the software which is proportional to the ratio of the perimeter squared over the radius squared) of the cells, with increasing values representing further deviation from cicular.

### BrdU incorporation

BMC-SMPCs were pulse-labelled by addition of BrdU (10 μM, Sigma) to the culture media for 24 h. After 10 d, cells were fixed in 4% paraformaldehyde (10 min, RT), permeabilized in 0.1% Triton X-100 (3 min, RT), and DNA was denatured by incubation in 2 N HCl (20 min, 37°C). After neutralization in borate buffer (0.1 M, pH 8.5), BrdU was detected with mouse monoclonal anti-BrdU IgG_1_ conjugated to Alexa 488 (2 μg/mL, clone PRB-1, Invitrogen), α-SMA was labelled as described above, and nuclei was counterstained with TO-PRO-3.

### Western Blot Analysis

BMCs, SMPCs, or aortic SMCs were lysed in lysis buffer (TBS containing 0.1% SDS, 0.02% NaN_3_, 100 μg/mL PMSF and 1 μg/mL aprotinin) for 20 min on ice, and cleared by centrifugation (14,000 *g*, 10 min, 4°C). Protein samples were separated by SDS-PAGE on 4–12% gradient gels and Western blots performed using α-SMA primary antibody (1 μg IgG_2a_κ/mL, clone 1A4, DAKO) and goat anti-mouse secondary antibody conjugated to horseradish peroxidase (1:2,000, Calbiochem).

### Preparation of fibrillar and non-fibrillar collagen coatings

Polymerized collagen fibrils were prepared from stock bovine dermal collagen I solution (Vitrogen, Cohesion Technologies Inc., CA) by dilution to 2.4 mg/mL with 10 X PBS, and the pH adjusted to 7.4. Non-fibrillar collagen solution was prepared by diluting the stock in 0.01 N HCl to a final concentration of 2.4 mg/mL. For matrix coating, collagen was incubated for a minimum of 1 h at 37°C to allow gelation (0.2 mL/cm^2^) and air-dried overnight. Coated surfaces were soaked in PBS or distilled H_2_O before use.

### Measurement of apoptotic cells by Annexin V and PI co-staining

BM-SMPCs or primary murine aortic SMCs were seeded at an initial density of 2.0 x 10^4^ cells/cm^2^ on TCPS coated with fibrillar or non-fibrillar collagen. Cells were treated with Annexin V staining solution (Roche) and Hoechst 33342 at 24, 48, or 72 h, (Molecular Probes), and the apoptotic index scored via fluorescence microscopy.

### Electron microscopy

BMCs were cultured for 10 d to allow differentiation and then seeded onto glass coverslips coated with non-fibrillar or fibrillar collagen. After 2 d, samples were incubated overnight with (0.1 M Na_2_HPO_3_, 0.0675 M NaOH, 1% glutaraldehyde, 3.7% formaldehyde, pH 7.3), postfixed for 30 min with 1% osmiumtetraoxide, and embedded in EPON^®^. Ultrathin sections (60–80 nm) were cut with an ultramicrotome, contrasted with uranyl acetate and lead citrate, and visualized using a JEOL JEM-1230 TEM transmission electron microscope.

### Statistical analysis

All data are presented as means (± SEM), where each *n* values represent data from an individual animal cell isolate. Multiple comparisons were assessed by ANOVA, followed by Tukey or Dunnett’s *post hoc* test for significance. When multiple hypotheses were tested simultaneously, significance levels were adjusted by the Bonferroni correction as specified.

## Results

### Smooth Muscle Differentiation of Bone Marrow Cells

Growth characteristics of early and late cultured bone marrow were compared by plating cells at 2.0 x 10^4^ cells/cm^2^ at 4 or 17 d after bone marrow isolation, and counting cell density over 3 to 4 d period. Early bone marrow cultures proliferated rapidly in 10% α-MEM, with a doubling time of 1.6 d. By 18 d, the projected population doubling time had lengthened to 5.1 d.

**Fig 1:**
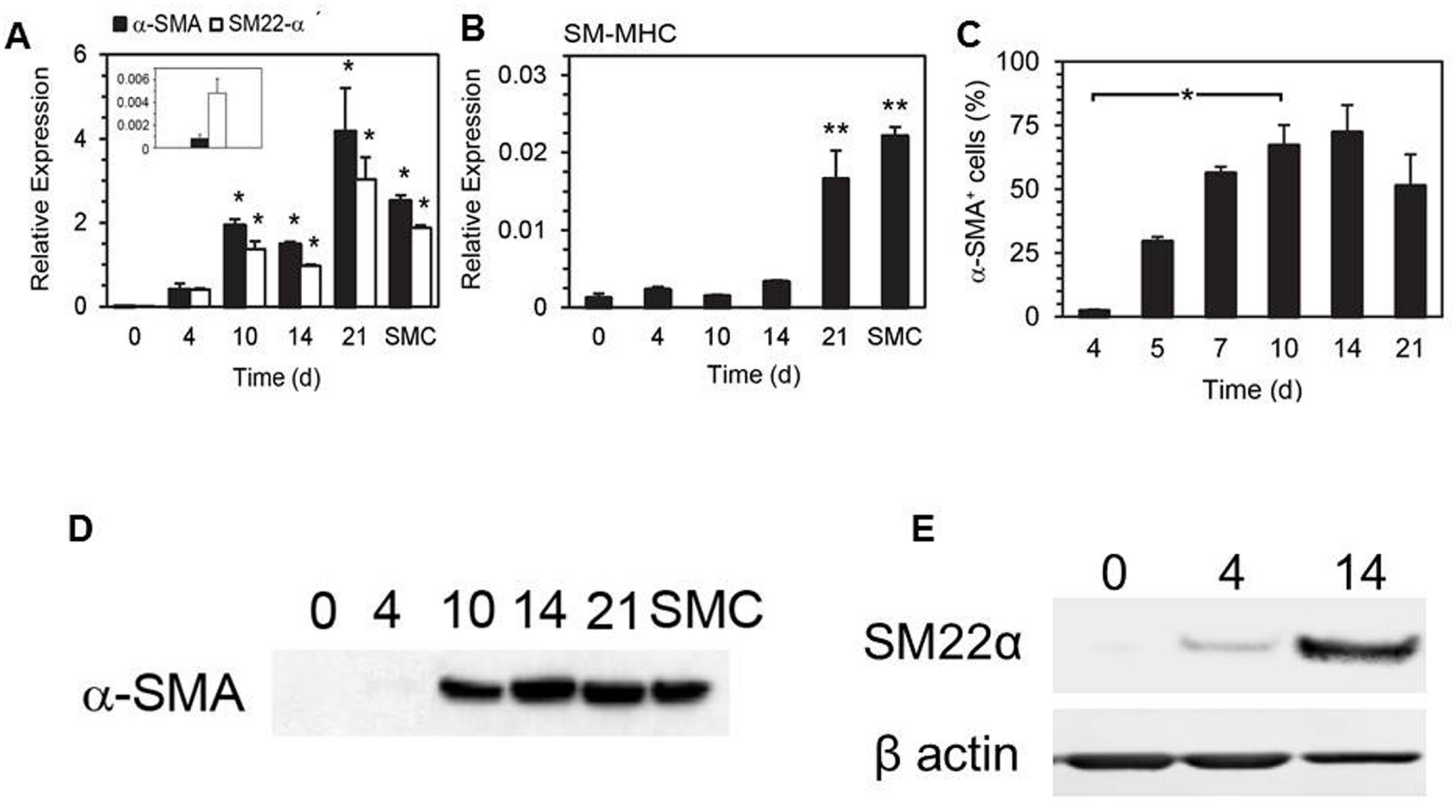
Bone marrow cells express smooth muscle cell markers upon short-term culture. qRT-PCR analysis of SMC marker expression in cultured bone marrow cells and murine aortic smooth muscle cells (SMCs) after the indicated culture period. Expression of α-SMA and SM22-α (A) and SM-MHC (B) was normalized relative to GAPDH (* *p* < 0.05, ** *p* < 0.01 compared to 0 d, Dunnett’s test, *n* = 3). The number of α-SMA^+^ cells was quantified for the indicated time (C). Western blot detection of α-SMA (D) and SM22-α (E) expression of mouse bone marrow cells cultured for the indicated period.

**Fig 2:**
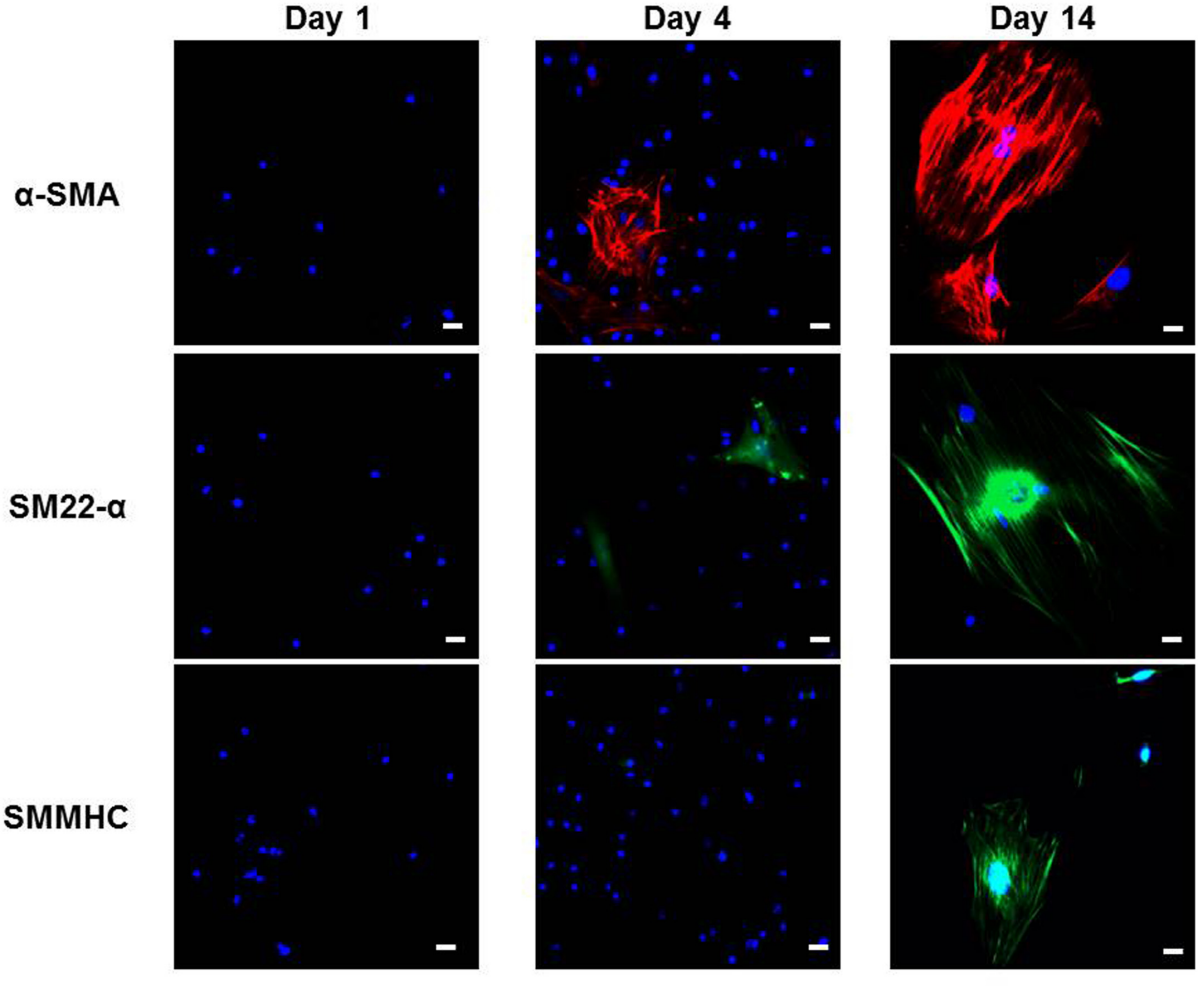
Bone marrow cells express smooth muscle cell markers at different time points. Immunofluorescence microscopy measurements of α-SMA+, SM22-α+, and SMMHC+ bone marrow cells in culture on days 1, 4, and 14. 20 X magnification, scale bar 20 μm.

Expression of SMC markers was quantified by qRT-PCR in bone marrow isolates over a 21 day period. In fresh bone marrow, α-SMA and SM22-α were not detected, after 10 d of culture in 10% α-MEM, a significant and sustained increase in expression of both α-SMA and SM22-α was detected (1.93 ± 0.15 fold relative to GAPDH and 1.50 ± 0.27 fold relative to GAPDH, respectively, *p* < 0.05, ANOVA using Dunnett’s *post hoc* test) (Fig 1A). Expression of SM-MHC was also significantly increased at 21 d in comparison to fresh bone marrow (0.017 ± 0.004 fold relative to GAPDH vs. 0.001 ± 0.001 fold relative to GAPDH at 0 d, *p* < 0.01, ANOVA using Dunnett’s *post hoc* test, *n* = 3) (Fig 1B). By 21 d, expression of α-SMA, SM22-α and SM-MHC in SMPCs had reached levels comparable to those in cultured murine aortic SMCs. The smooth muscle differentiation was examined at the protein level by Western Blot and Immunocytochemical analysis of α-SMA, SM22-α, and SM-MHC (Figs 1D, 1E and 2). Although α-SMA, SM22-α, and SMMHC were not detected in fresh BMCs, by 14 days their abundance was markedly increased. The percentage of cells expressing cytoskeletal α-SMA underwent a rapid increase during early culture, rising from 2.3 ± 0.5% at 4 d to 67.2 ± 8.0% at 10 d (*p* < 0.01, Bonferroni multiple comparisons test, *n* = 3) (Fig 1C).

### Proliferation of α-SMA^+^and α-SMA^-^ BMCs

**Fig 3:**
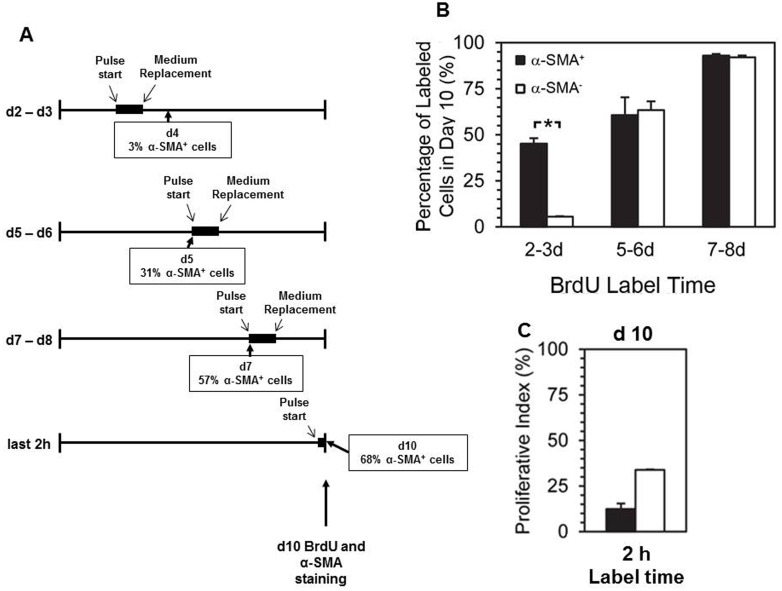
Cells committed to SMPC lineage in early bone marrow cultures are marked by a high proliferative index. BMCs were pulse-labelled with BrdU for a 24 h period starting at days 2, 5, and 7 and subsequently immuno-stained for BrdU and α-SMA at day 10, a subgroup was labelled with BrdU for 2 hours immediately prior to staining, percentages of α-SMA^+^ cells during culture periods are indicated (A). At day 10 cells were stained for BrdU and α-SMA, the percentage of α-SMA^+^ and α-SMA^-^ cells labelled with BrdU during the indicated time points are plotted (B and C) (*p < 0.05, Bonferroni multiple comparisons test, *n* = 3).

BMCs were pulse-labelled (24 hr) with BrdU at 2, 5, or 7 d and for 2 hours at 10 d after plating, and all cells were immunostained for α-SMA and BrdU at 10 d (Fig 3A). The proportion of cells that were proliferating during each pulse-label was compared between SMPCs (α-SMA^+^ at 10 d) and other BMCs (α-SMA^-^ at 10 d) (Fig 3B and 3C). Cells committed to SMPC differentiation were highly proliferative in early culture, and their proliferative index increased to 90% by 7 d. α-SMA^-^ BMCs initially displayed very low proliferation (3% BrdU^+^ cells at 2 d) but this increased at later time points, reaching 60% by 5 d and 90% by 10 d. In early cultures, the difference between proliferative indices in cells differentiating to SMPCs versus not differentiating was highly significant (45.5 ± 5.5% vs. 3.0 ± 0.3% at 2 d, *p* < 0.01, Bonferroni multiple comparisons test).

### SMPC differentiation is modulated by PDGF-BB

**Fig 4:**
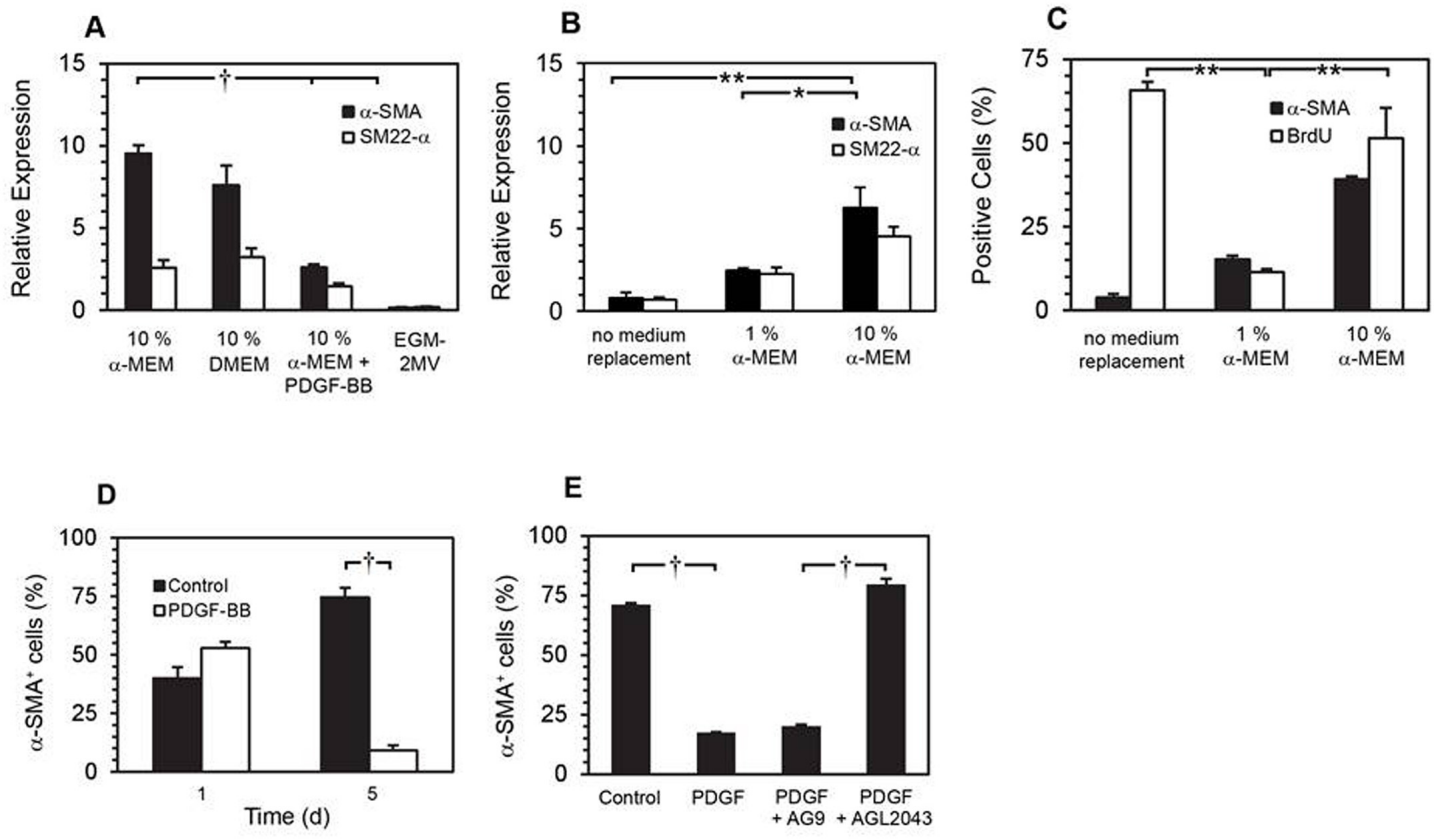
Effects of PDGF-BB on smooth muscle cell marker expression. qRT-PCR analysis of α-SMA and SM22-α expression in BMCs cultured for 10 d in the specified media (A) († *p* < 0.001, Tukey-Kramer multiple comparisons test, *n* = 3). All BMCs were cultured in 10% α-MEM with additional PDGF-BB (50 ng/mL) for 8 d before replacing the media as indicated for 2 d. qRT-PCR analysis of α-SMA and SM22-α expression at 10 d, normalized against GAPDH (B). (* *p* < 0.05, ** *p* < 0.01, Bonferroni multiple comparisons test, *n* = 3). At 10 d, cells were immunolabelled for α-SMA and BrdU (** *p* < 0.01, Bonferroni multiple comparisons test, *n* = 3) (C). SMPCs were re-plated at day 10 in 10% α-MEM ± supplemental PDGF-BB (50 ng/mL) for 1 and 5 days, followed by immunofluorescence labelling of α-SMA (D) († *p* < 0.001, Bonferroni multiple comparisons test, *n* = 3). SMPC culture media was replaced with: control (no media change), media plus PDGF-BB (50 ng/mL), media plus PDGF-BB (50 ng/mL) and AG9 (10 μM), media plus PDGF-BB (50 ng/mL) and AGL2043 (10 μM), as indicated. Expression of α-SMA was detected by immunofluorescence microscopy after 5 d incubation (E) († *p* <0.001, Tukey-Kramer multiple comparisons test, *n* = 3).

BMCs were cultured in 10% α-MEM, 10% DMEM, 10% α-MEM + PDGF-BB (50 ng/mL) or EGM-2MV for 10 d before qRT-PCR analysis of α-SMA and SM22-α gene expression. At 10 d, robust expression of α-SMA and SM22-α was observed in SMPCs cultured in 10% α-MEM or 10% DMEM, but not in EGM-2MV (an endothelial growth medium used as a negative control). When PDGF-BB (50 ng/mL) was added to 10% α-MEM media, significantly less α-SMA expression was observed (Fig 4A, α-SMA, 3.6-fold less, *p* < 0.001, Tukey-Kramer multiple comparisons test).

In order to test whether suppression of SMC markers by PDGF-BB was reversible, 10% α-MEM + PDGF-BB culture media was replaced at day 8 with α-MEM containing 1 or 10% FBS, and expression of α-SMA and SM22-α was analyzed at day 10. qRT-PCR analysis revealed that α-SMA and SM22-α expression was rapidly restored after PDGF-BB containing media was replaced with 10% α-MEM (Fig 4B). In BMCs cultured in 10% α-MEM + PDGF-BB 10d (control) we observed suppression of α-SMA and high cell proliferation yet replacing the media for 2 d with 10% MEM alone restored α-SMA expression (39.1 ± 0.9%), even though cell proliferation remained high (65.7 ± 2.4% BrdU^+^), replacing the media with 1% MEM suppressed both α-SMA and cell proliferation (Fig 4C).

The inhibitory effect of PDGF-BB on α-SMA expression was further examined in differentiated SMPCs (10 d culture in 10% α-MEM), no significant change was observed after 24 h treatment, but after 5 d incubation with supplemental PDGF-BB (50 ng/mL) the percentage of α-SMA^+^ cells was significantly lower (74.5 ± 4.2% vs. 9.2 ± 2.0%, *p* < 0.001, Bonferroni multiple comparisons test, *n* = 3, Fig 4D). This α-SMA suppression was receptor dependent as demonstrated by culturing SMPCs in the presence of exogenous PDGF-BB (50 ng/mL) and a PDGFR-selective tyrosine kinase inhibitor (AGL2043, 10 μM) or a negative control compound AG9 (10 μM) for 5 d (79.5 ± 2.5 vs 18.3 ± 0.6%, respectively, *p* < 0.001, Tukey-Kramer multiple comparisons test, *n* = 3).

### Effects of collagen substrate on SMPC growth and viability

**Fig 5:**
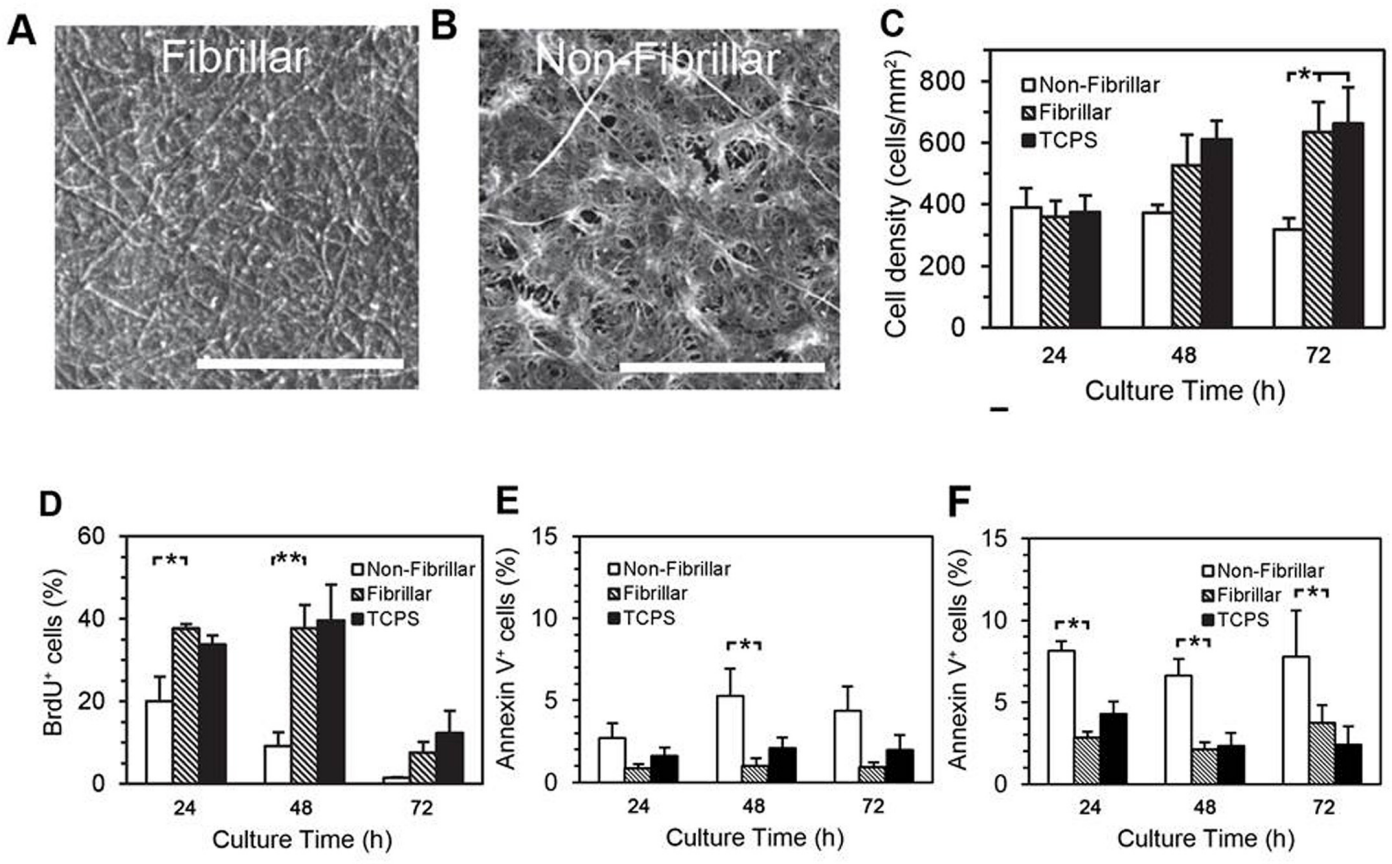
Effects of collagen polymerization on SMPC proliferation and viability. Representative SEM images depicting the topology of surfaces coated with fibrillar (A) or non-fibrillar collagen preparations (B, scale bars are 10 μm). BMCs cultured for 10 d in 10% α-MEM, and then re-plated onto substrates as indicated, at 24, 48 and 72 h, cell density (C) and BrdU incorporation (D) were measured. Indices of Apoptosis were assessed in SMPCs (E) and primary cultures of murine SMCs (F) by Annexin V/PI co-staining. (* *p* < 0.05, ** *p* < 0.01, (C, D, *n* = 4), (E, F, *n* = 3), Tukey-Kramer multiple comparisons test).

The topography of the collagen coatings was examined by scanning electron microscopy (SEM), and revealed an even coating of collagen fibrils 100 nm wide, reaching lengths upwards of 10 μm in the polymeric (fibrillar) gels (Fig 5A); in contrast the non-fibrillar coatings were distributed in a mesh-like network (Fig 5B). TEM of the acidic collagen solution confirmed the presence of 250 nm by 10 nm rod-like structures, consistent with the expected dimensions for collagen monomers (data not shown). Collagen coating thicknesses of approximately 15 μm were measured on transverse sections by TEM.

When 10d SMPCs were re-plated onto the collagen gels numbers increased on fibrillar collagen and uncoated TCPS after 48 and 72 h (non-fibrillar vs. fibrillar substrate, 319 ± 36 vs. 635 ± 97 cells/mm^2^ at 72 h, respectively, *p* < 0.05, Bonferroni multiple comparisons test, *n* = 4) (Fig 5C), yet on non-fibrillar collagen SMPC failed to grow over the 72 h period. Pulse-labelling of 10 d SMPCs with BrdU (10 μM, 24 h) confirmed that SMPC proliferation on non-fibrillar collagen was significantly less than that observed on fibrillar collagen or on TCPS (Fig 5D) (non-fibrillar vs. fibrillar collagen, *p* < 0.05 at 24 h, *p* < 0.01 at 48 h, Bonferroni multiple comparisons test, *n* = 4). Annexin V staining demonstrated a small but significant increase in the number of apoptotic SMPCs on non-fibrillar collagen, compared to fibrillar collagen and TCPS (5.3 ± 1.6% vs. 1.0 ± 0.5% at 48 h (fibrillar), *p* < 0.05, Bonferroni multiple comparisons test, *n* = 4) (Fig 5E). Similarly, apoptosis was increased in primary aortic SMCs plated on non-fibrillar collagen (Fig 5F).

### Impact of collagen substrate on SMPC adhesion and differentiation

**Fig 6:**
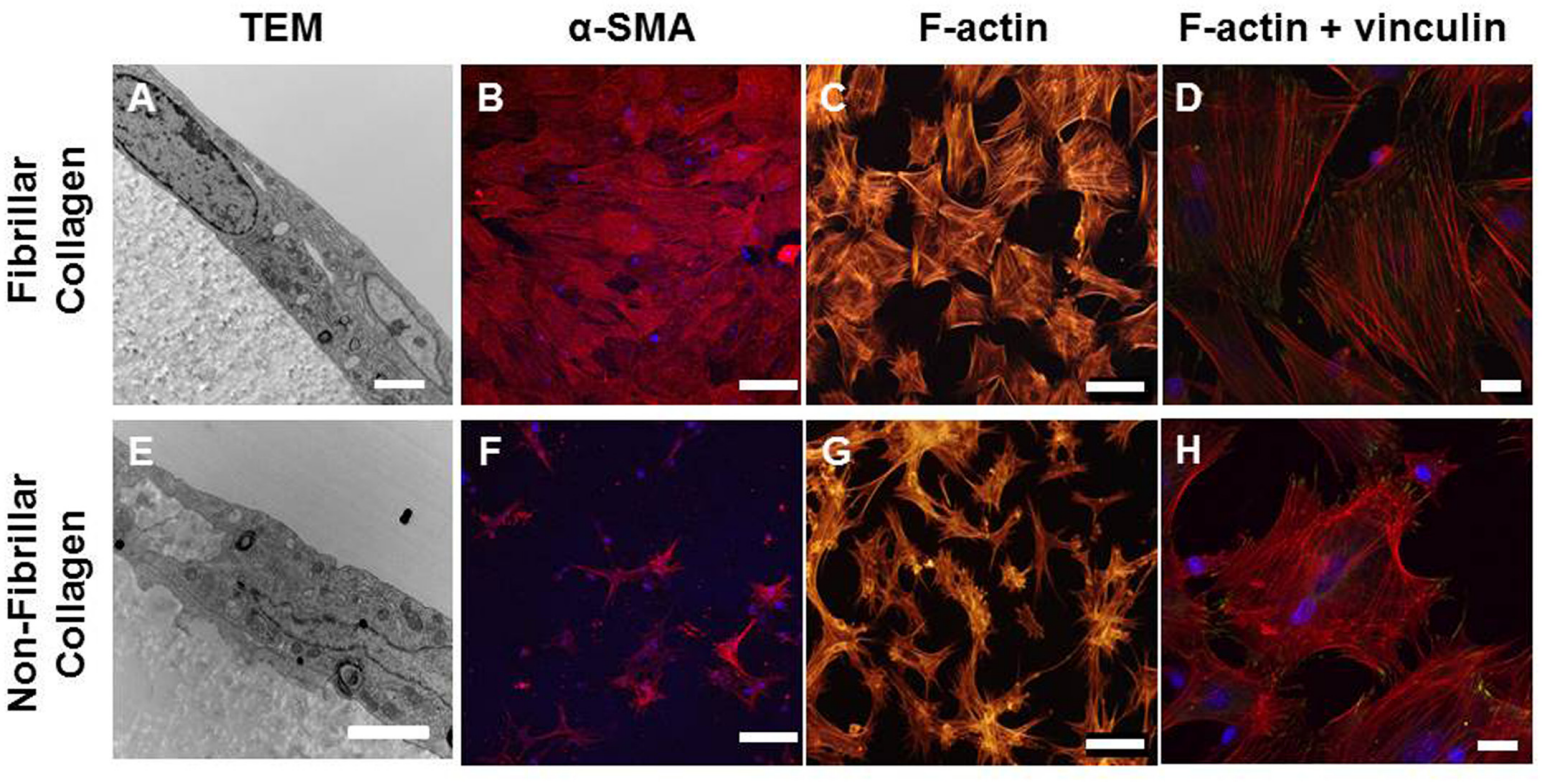
SMPC differentiation and focal adhesion formation are suppressed on monomeric collagen. Bone marrow cells were cultured for 10 d in 10% α-MEM media on TCPS to allow expression of SMC markers and re-plated onto fibrillar or non-fibrillar collagen for 72 h. (A, E) TEM images of transverse sections through SMPCs on collagen matrices. Magnification, (A) 10,000 X; (E) 15,000 X. Scale bars 2 μm. (B, F) Representative images for SMA expression at 72 h. (C, G) Phalloidin-Alexa 546 labelling of SMPC F-actin. Magnification, 20 X. Scale bars, 100 μm. (D, H) Co-labelling with phalloidin and mouse anti-vinculin antibody. 60 X magnification, scale bars 20 μm.

We performed a microscopic comparison of the attachment of SMPCs to fibrillar and non-fibrillar collagen. After 48 h of culture on collagen, TEM images showed uninterrupted contact between the basal membranes of SMPCs and fibrillar collagen-coated surfaces (Fig 6A). On non-fibrillar collagen, however, only discrete attachment points were observed, and were interspersed with gaps up to 500 nm wide between SMPCs and the substrate (Fig 6E).

The apparently reduced attachment of SMPCs to non-fibrillar collagen was also associated with changes in focal adhesion contacts. SMPC stress fibres and focal adhesions were detected by phalloidin and vinculin co-labelling. After 48 h culture on fibrillar collagen, SMPCs displayed a polygonal morphology with focal adhesion contacts dispersed across the basal surface (Fig 6C and 6D). On non-fibrillar collagen, SMPCs had a stellate appearance and a limited number of focal adhesion contacts were restricted to the periphery of the cell (Fig 6G and 6H). The number of focal adhesion sites per cell was three fold higher in cells cultured on fibrillar as opposed to monomeric collagen.

**Fig 7:**
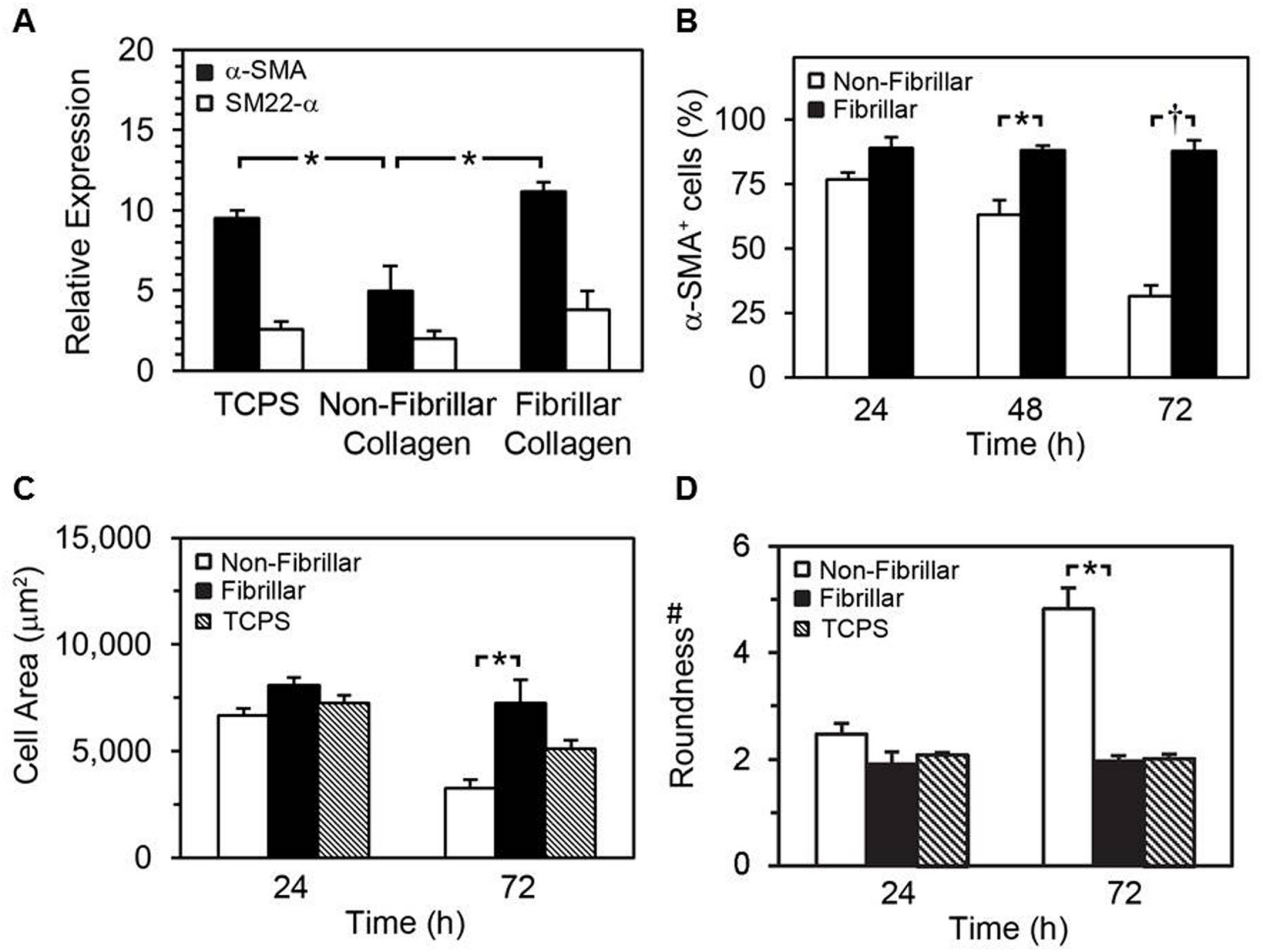
Effects of monomeric collagen on SMPC differentiation and cell shape. qRT-PCR analysis of expression of α-SMA and SM22-α mRNA in BMCs cultured for 10 d on the indicated substrates (A) (* *p* < 0.01, Bonferroni multiple comparisons test, *n* = 3). Bone marrow cells were cultured for 10 d in 10% α-MEM media on TCPS followed by re-plating on indicated collagen surfaces for 24 to 72 h. Quantification of α-SMA^+^ cells at indicated period via immunofluorescence microscopy using mouse anti-αSMA antibody and DAPI (B).(* *p* < 0.05, † *p* < 0.001, Bonferroni multiple comparisons test, *n* = 3). Morphometric analysis of cell area (C) and roundness (D) after culture for 24 and 72 h on collagen was achieved. #: roundness is proportional to the ratio of the perimeter squared over the radius squared of the cells, with increasing values representing further deviation from circular.

To investigate whether acquisition of the SMPC phenotype was influenced by interactions with substrate collagen, BMCs were cultured on collagen immediately after isolation, and expression of α-SMA and SM22-α was measured by qRT-PCR after 10 d. In line with the trace expression levels of α-SMA and SM22-α previously detected by qRT-PCR in fresh BMCs (Fig 1), expression of both genes was stimulated after 10 d of culture on fibrillar collagen or TCPS (Fig 7A). On non-fibrillar collagen, the stimulatory effect on α-SMA expression was reduced compared to fibrillar collagen (6.1 ± 0.6 compared to 11.2 ± 0.6 fold relative to GAPDH, *p* < 0.01, Bonferroni multiple comparisons test, *n* = 3).

Lastly, we measured the effects of the collagen substrate on differentiation in established SMPCs by quantifying the percentage of α-SMA-expressing cells during a 72 h period of culture (Fig 7B). Expression of α-SMA was robust and sustained in SMPCs grown on fibrillar collagen. On non-fibrillar collagen, the number of α-SMA^+^ cells progressively decreased, reaching 31.6 ± 4.2% by 72 h (vs. 87.7 ± 4.3 for fibrillar collagen, *p* < 0.001, Bonferroni multiple comparisons test, *n* = 4). In addition, significant differences in SMPC area and roundness were found upon morphometric image analysis after 72 h culture on collagen, (Fig 7C and 7D).

## Discussion

Vascular smooth muscle-like cells have been previously derived from a number of extra-arterial sources including peripheral blood and bone marrow and it has been speculated that these cells may play a role in vascular repair or disease progression (reviewed in [29, 30]). However, the subpopulation of smooth muscle progenitor cells in the bone marrow stroma or the conditions driving smooth muscle differentiation have not been fully described. Kashiwakura et al transfected bone marrow stromal cells (BMSCs) with an SM22α promoter-GFP construct and demonstrated that less than 3% of BMSCs stably expressed SM22α at 10 days of culture and these cells went on to express PDGFβR, α-SMA, calponin, and SM-MHC at later time points [31]. Metharom et al have demonstrated that the CX_3_CR_1_-expressing cells in murine bone marrow (representing approximately 10% of the cell population) contain the smooth muscle progenitor population and it is these CX_3_CR_1_-expressing cells that are involved in the response to vascular injury [27, 32]. In agreement with these previous studies, we found a population of bone marrow cells with a propensity for differentiation towards the smooth muscle cell phenotype; however, unlike Kashiwakura et al we found expression of α-SMA was markedly induced in 67 ± 1% of the cells within 10 days [31]. By 14 days α-SMA, SM22- α, and SM-MHC were all detectable by immunohistochemistry and by 21 days the mRNA expression of SM-MHC was robust, suggesting that cells reach mature SMC phenotype. In our cultures the differentiated SMCs were largely derived from a subset of cells with a high rate of proliferation between day 2 and day 3 after bone marrow plating, suggesting that SMC progenitors can be identified early (by proliferation) and amplified before the appearance of smooth muscle markers (3% α-SMA on day 4). This early proliferation of smooth muscle progenitors may be distinct to our culture conditions and in part explain the more rapid appearance of the smooth muscle cell phenotype. Serum contains a number of components that may, at high concentrations, act as inhibitors for smooth muscle cell growth [33–35] in our study, we used 10% FBS which is much lower than 25% used by Kashiwakura et al [31] and as such may be more favourable for early SMC progenitor proliferation.

PDGF-BB, a potent mitogenic factor in SMCs, is a component in serum (PDGF-BB levels in human serum range from 2.19–10.67 ng/mL, [36, 37]) and has been shown to suppress expression of α-SMA, SM-MHC and SM α-tropomysin in mature SMCs [19, 20, 38]. In our hands supplementing the cultures with additional PDGF markedly suppressed expression of several key markers of smooth muscle cell differentiation, this effect was reversed within 2 days of PDGF withdrawal and was specifically blocked by the PDGFR tyrosine kinase inhibitor AGL2043. The suppression of α-SMA by PDGF was not linked to enhanced cellular proliferation as proliferation rates were similar in cells showing high α-SMA expression supplemented with 10% FBS. It is possible that the high levels of PDGF simply repress expression of key SMC markers and not complete differentiation to the SMC phenotype. The repression of α-SMA by PDGF-BB is consistent with the findings of Dandre et al [39] for sub-confluent cultures of arterial derived smooth muscle cells. Similar to our studies Banai et al used the selective PDGFRβ inhibitor AG1295 on carotid out growth smooth muscle cells showing 5 days of treatment markedly enhanced levels of α-SMA [40]. Regulation of cell differentiation and marker expression may depend on the proportional activation of the PDGF receptors alpha or beta, which differentially regulate RhoE and Rho-associated kinase (ROCK) activity and subsequently cytoskeletal α-SMA depolymerisation [41].

Fibrillar collagen is a major component of the vascular extracellular matrix yet in pathological conditions, excessive degradation and or synthesis of collagen can lead to the accumulation of non-fibrillar forms (reviewed in [42]). Non-fibrillar collagen has been previously shown to reduce SMC contractility, stimulate inflammatory protein expression such as VCAM-1 and NF-kappaB and inhibit SMC differentiation [15, 19]. In this study we produced two collagen substrates by either allowing bovine dermal collagen to polymerize at neutral pH or gelling the collagen under acidic conditions, by this method we produced a substrate with a detectable fibre structure or one principally deplete of fibres (Fig 5A and 5B and S1 Fig). We observed that the proliferation of SMPCs was markedly reduced and apoptosis increased on non-fibrillar compared to fibrillar collagen. In contrast Koyama et al demonstrated that polymerized fibrillar collagen arrests adult smooth muscle cell proliferation while monomeric collagen supports cell division[17]. These opposing results may be due to the differences in cell types, SMPCs versus adult SMCs in the two studies, or more likely due to differences in the collagen substrates. In our study we used gels with a defined thickness as opposed to the thin coatings employed by Koyama et al, when we used similar thin coatings we also observed a significant decrease of proliferation in SMPCs on fibillar collagen compared to non-fibrillar collagen (data not shown). Hydroxyproline assays used to quantify collagen content after coating demonstrated Koyama’s method (specifically for non-fibrillar collagen) resulted in little protein deposition (S1 Fig) suggesting that the cell response to these very thin coatings are likely influenced by the rigid plastic substrate as has been demonstrated for hMSCs [43]. Rigid substrates support enhanced smooth muscle cell proliferation [44] and differentiation [45] from bone marrow-derived MSCs compare to soft surface which is similar to the response to non-fibrillar collagen seen in Koyama’s study. We believe that the thicker collagen gels more closely represent the three-dimensional scaffold present in the vascular wall.

Differentiation of the bone marrow cells to a smooth muscle phenotype was markedly reduced when the cells were initially plated on non-fibrillar collagen, although this result may also reflect an increase loss of smooth muscle committed cells on this substrate as apoptosis of differentiated SMPCs was higher on non-fibrillar collagen. In agreement with a previous study [19] on smooth muscle cells, we observed that the expression of α-SMA in SMPCs was rapidly suppressed when the cells were re-plated on non-fibrillar collagen yet fibrillar collagen supported the maintenance of SMC marker expression. These shorter term passage studies may better reflect the response to the defined substrate as it is our experience that matrix deposition by SMCs is very limited over a 72 hour time period (S2B and S2C Fig), nor is it likely that substantial matrix is transferred by the cells after dissociation with trypsin [46]. Thus fibrillar collagen substrates appear to be much more favourable to SMPC survival and differentiation and collagen structural changes may play a key role in the vascular response to injury.

In studies with human aortic SMCs Guildford et al demonstrated a maintenance of contractile phenotype with cells grown on a collagen rich matrix and exposed to PDGF [47], whereas Chen et al demonstrated that human aortic SMCs grown on polymerized collagen show high levels of contractile markers (α-SMA, myosin heavy chain) and that expression of these markers is suppressed by the combination of PDGF-BB and IL-1β [19]. In contrast the murine SMPCs and adult aortic SMCs cells used in our studies were responsive to PDGF-BB alone and this may reflect differences in intracellular signalling between the human and murine cell types. A later study by the Chen group has demonstrated the marked utility of polymerized collagen in the derivation of SMCs from human placental-derived multi-potent cells confirming a critical role for the p38 MAPK pathway and serum response factor in matrix induced differentiation and maintenance of SMC phenotype [48]. Taken together these results suggest a subtle balance between the positive effects of a fibrillar matrix and those of PDGF stimulation in the maintenance of a regenerative SMC phenotype in the vascular wall.

Our results confirm that smooth muscle-like cells may be derived from a bone marrow origin, and highlight the critical importance of their interactions with the surrounding matrix. The high levels of PDGF-BB and monomeric collagen, associated with some atherosclerotic plaques, may restrict the differentiation and subsequent survival of SMPCs in the vessel wall. The role of SMPCs in vascular disease has not been fully defined yet appropriate healing in response to vascular injury may be enhanced by therapies designed to enhance SMPC differentiation and survival at the site of injury. In this context, SMPC response to PDGF-BB and collagen structure, in particular, are promising targets for further study.

## Supporting Information

S1 Fig Collagen fibre structure and content after coating. TEM images of fibrillar collagen (A). D banding structure was indicated by black arrows. Scale bar indicates 500 nm. The average length of indicated D banding is 18.5 ± 0.2 nm. The collagen content of different coatings was assessed by quantifying their hydroxyproline content. Different methods of collagen coating were indicated. For each type of coating, three preparations were assessed: A) no rehydration, B) rehydration, and C) rehydration with 24 hours cell growth.
(TIF)

S2 Fig Studies on human aortic smooth muscle cells (HASMCs). Cells were plated on non-fibrillar or fibrillar collagen coated surfaces with or without PDGF-BB (50ng/mL) treatment. At days 1 and 5, percentage of α-SMA positive cells was determined in each culture (A). n = 3. ** and *** indicate p<0.01 and p<0.001, respectively. HASMCs were cultured on TCPS for 72 hours followed by staining with vinculin antibody and phalloidin (B) or decellularization and staining with collagen I antibody (C). Scale bar indicates 20 μm.
(TIF)
