# Supplementary figures and images for: Differentiation of Murine Bone Marrow-Derived Smooth Muscle Progenitor Cells Is Regulated by PDGF-BB and Collagen
Source: PLoS One. 2016 Jun 3;11(6):e0156935. doi: 10.1371/journal.pone.0156935 (PMC4892566; doi:10.1371/journal.pone.0156935)

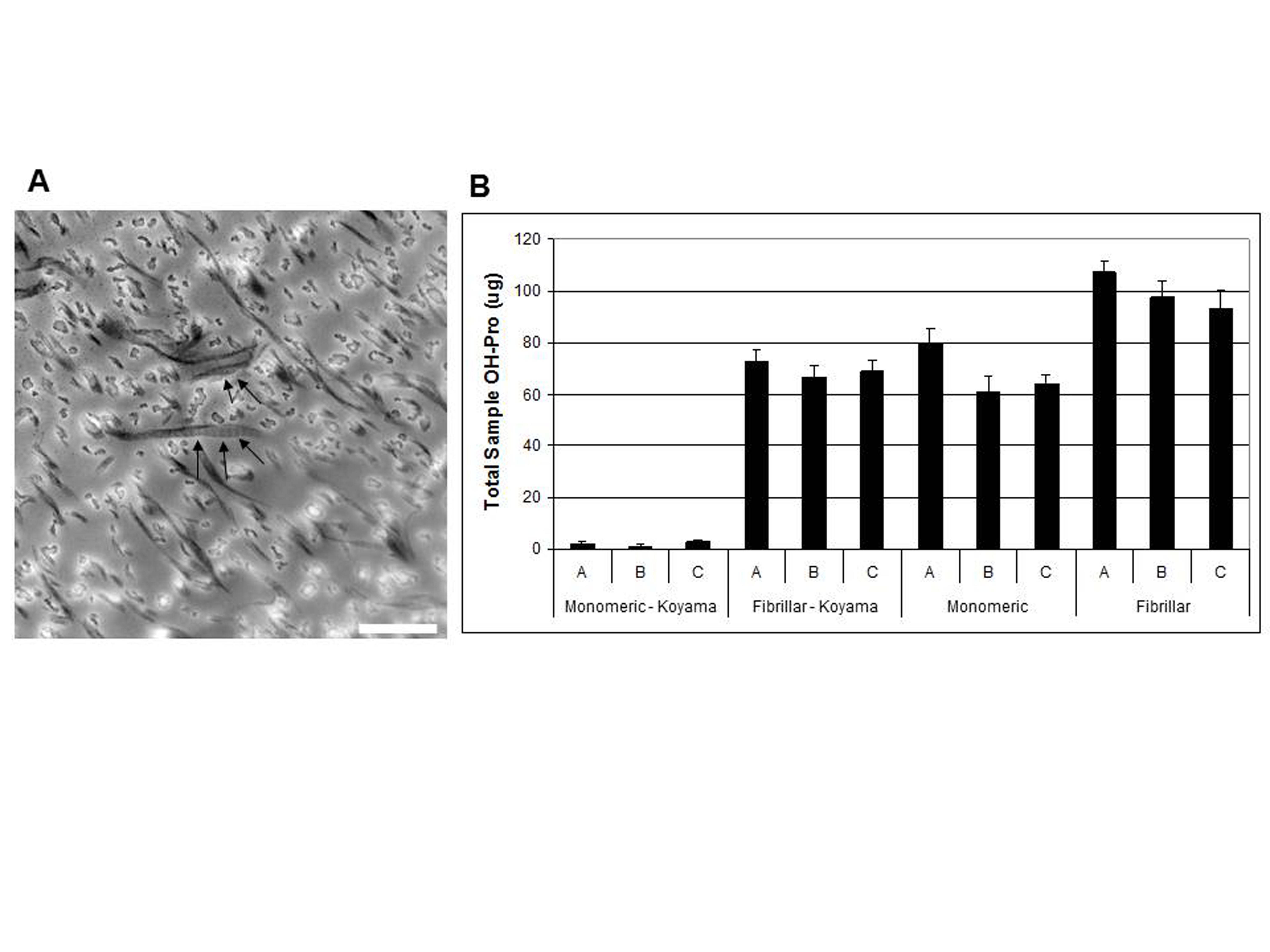

Supplement: S1 Fig — TEM images of fibrillar collagen (A). D banding structure was indicated by black arrows. Scale bar indicates 500 nm. The average length of indicated D banding is 18.5 ± 0.2 nm. The collagen content of different coatings was assessed by quantifying their hydroxyproline content. Different methods of collagen coating were indicated. For each type of coating, three preparations were assessed: A) no rehydration, B) rehydration, and C) rehydration with 24 hours cell growth. (TIF) [file pone.0156935.s001.tif]

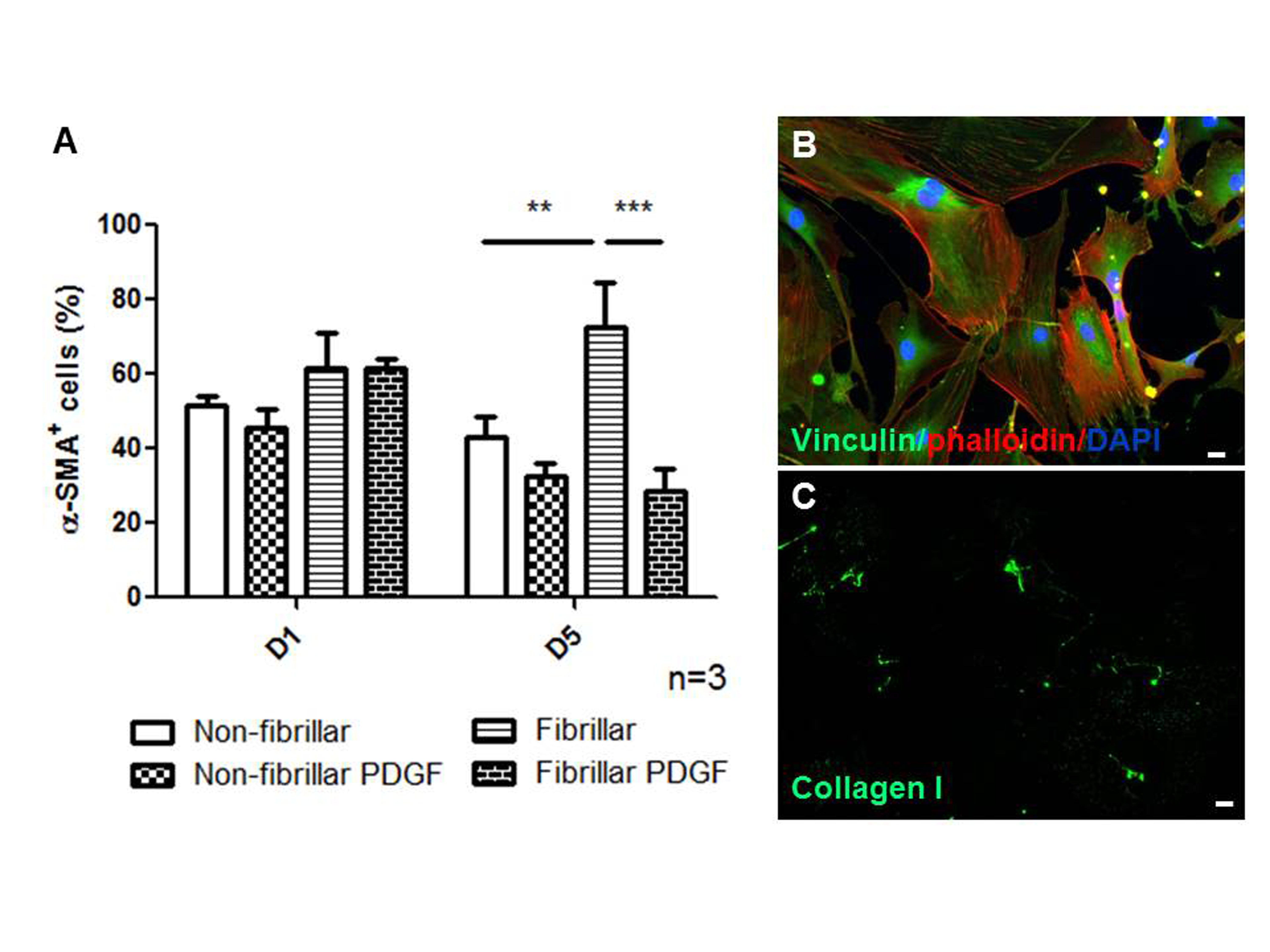

Supplement: S2 Fig — Cells were plated on non-fibrillar or fibrillar collagen coated surfaces with or without PDGF-BB (50ng/mL) treatment. At days 1 and 5, percentage of α-SMA positive cells was determined in each culture (A). n = 3. ** and *** indicate p<0.01 and p<0.001, respectively. HASMCs were cultured on TCPS for 72 hours followed by staining with vinculin antibody and phalloidin (B) or decellularization and staining with collagen I antibody (C). Scale bar indicates 20 μm. (TIF) [file pone.0156935.s002.tif]
